# Auditory Delta Event-Related Oscillatory Responses are Decreased in Alzheimer’s Disease

**DOI:** 10.3233/BEN-2012-0344

**Published:** 2011-12-29

**Authors:** G. G. Yener, B. Güntekin, D. Necioglu Örken, E. Tülay, H. Forta, E. Başar

**Affiliations:** ^1^Departments of Neurology and Neurosciences; Dokuz Eylül UniversityIzmirTurkey; ^2^Brain Dynamics and Multidisciplinary Research CenterDokuz Eylül UniversityIzmirTurkey; ^3^Brain DynamicsCognition and Complex Systems Research CenterIstanbul Kultur UniversityIstanbulTurkey; ^4^Sisli Etfal State HospitalDepartment of NeurologyIstanbulTurkey

**Keywords:** Alzheimer, P300, oscillations, auditory, delta, cholinergic

## Abstract

*Background:* Visual delta event-related (ERO) and evoked oscillations (EO) of Alzheimer patients (AD) are different than healthy. In the present study, the analysis is extented to include auditory ERO and EO in AD. The rationale is to reveal whether the auditory ERO delta responses are also reduced, and whether this is a general phenomenon in Alzheimer patients upon applying stimuli with cognitive load.

*Methods:* Thirty-four mild AD subjects (17 de-novo and 17 medicated (cholinergic)) and seventeen healthy controls were included. Auditory oddball paradigm and sensory auditory stimuli were applied to the subjects. Oscillatory responses were analyzed by measuring maximum amplitudes in delta frequency range (0.5–3.5 Hz).

*Results:* Auditory delta ERO (0.5–3.5 Hz) responses of healthy controls were higher than either de-novo AD or medicated AD group, without a difference between two AD subgroups. Furthermore, the auditory EO after presentation of tone bursts yielded no group difference.

*Conclusion:* Our findings imply that delta ERO is highly unstable in AD patients in comparison to age-matched healthy controls only during the cognitive paradigm. Our results favor the hypothesis that neural delta networks are activated during cognitive tasks and that the reduced delta response is a general phenomenon in AD, due to cognitive impairment.

Abbreviations: ERO: Event-related oscillations, EO: Evoked oscillations, AD: Alzheimer's disease, EEG: Electroencephalography, MMSE: The Mini-mental State Examination

## 1. Introduction

Alzheimer type dementia (AD) is one of the leading neurological conditions most responsible for neuropsychiatric morbidity in elderly individuals. The hallmark lesions of AD are amyloid plaques and neurofibrillary tangles. Clinical symptoms closely relate to tangles, neurodegeneration, and synapse loss. Clinical signs of AD are characterized by progressive cognitive deterioration, together with declining activities in daily life and by cognitive and neuropsychiatric symptoms [42].

Transmission of information in the nervous system is provided by electrical impulses between neurons. Oscillatory electrical activity is the most obvious observable type of electrical activity in the brain. It is now possible to achieve measurements, via scalp electrodes, of human subjects under various states of behavior and learning, sensory or cognitive events. Spontaneous electroencephalography (EEG) is widely studied for cognitive disorders in clinical practice [1,48]. Event-related oscillations (ERO) provide a powerful technique, with high temporal resolution, which can

ISSN 0953-4180/12/$27.50 © 2012 – IOS Press and the authors. All rights reserved be used as a tool for detecting subtle abnormalities of cognitive processes [6,7,40]. ERO is elicited by digital filtering of P300 that occurs upon application of a cognitive task, the oddball paradigm. Early experimental studies on large scale brain activity in animals and humans showed superposition of multiple oscillations in delta (0.5–3.5 Hz), theta (3.5–7 Hz), alpha (8–13 Hz), beta (18–30 Hz), gamma (30–70 Hz) and higher frequencies as the 300 Hz component in various parts of the brain [6]. These oscillatory systems are selectively distributed in the brain. They control the integrative brain functions at all sensory and cognitive levels [7].

It has been well known for several decades that P300 is attenuated in AD. The full potential of electrophysiological methods in helping to predict [2,14,35], to diagnose [34,24,26], and to monitor either treatment or progress [38,47] in AD patients has not been reflected in routine clinical practice. These techniques have the potential to investigate changes in brain dynamics. In our previous work, we explored visual event-related oscillations and evoked oscillations (EO) in AD [43, 44,47]. The term "event-related" is used for a potential elicited after an event including cognitive task. On the other hand, the expression "evoked" is used when the potential is elicited by simple sensory stimulation. The term "oscillation" implies rhythm within a specific time interval. These oscillations following the stimulation are considered as real brain responses [5]. ERO in various frequency ranges may reflect different aspects of information processing [6,7]. Even though different frequencies are related to a wide variety of cognitive functions [27,39,41], correlations between oscillatory frequency bands and some cognitive functions have been reported. For example, alpha oscillatory responses increase with simple memory tasks [30], and beta oscillatory responses are important in facial expression [21] or after negative emotion stimulus in humans [22]. Theta ERO responses have been proposed to be related to physiological memory processes [25,29]. Also theta responsiveness in frontal lobes is interpreted as an indication of the function of the hippocampo-fronto-parietal system during cognitive processes [17] whereas delta responses are considered to be related to decision-making [8]. In our recent clinical studies on bipolar disorders and AD, difference from control subjects were observed in specific frequencies. Event-related alpha and delta oscillatory responses are greatly reduced respectively in subjects with bipolar disorder and AD, possibly reflecting cognitive changes seen in these disorders [33,46]. In subjects with Parkinson's disease [37] or schizophrenia [9], lower values of event-related theta oscillatory responses are found than controls, indicating that these oscillations appear to be involved in mnemonic networks.

Our earlier studies included comparison of visual evoked and event-related responses in AD. By investigating visual event-related oscillations, we found that de-novo AD group had lower theta phase-locking than controls at left frontal electrodes and cholinergic treatment increased phase-locking in this range, similar to controls [47]. Moreover, in these experiments we also found decreased maximum amplitudes of visual event-related delta responses in AD subjects regardless of cholinergic medication [43]. Visual event-related coherences were decreased between frontal and almost all other parts of brain in AD, indicating a widespread cortico-cortical disconnection in this disease [3,4]. In addition, visual evoked coherences that are elicited after simple sensory stimulations were significantly decreased between only fronto-occipital electrode sites in the same patient group [23].

Contrary to general acceptance, the evoked delta oscillatory responses where no cognitive task was involved showed an increase over posterior parts of the brain in AD subjects; on the other hand, event-related delta oscillatory responses had diminished values over fronto-central areas in AD [44]. This differing results after cognitive and sensory stimuli implied that there were at least two different networks, depending on the cognitive load of stimuli. Moreover, inhibition of posterior parts of the brain by prefrontal cortices may be the cause of the phenomenon mentioned above [11].

The question of whether the same brain dynamics prevail in the auditory system, still remains to be clarified. An earlier study indicates decreased event-related delta oscillatory responses in AD [13]. However no previous study has compared brain auditory evoked and event-related delta oscillatory responses. Investigating these oscillations by means of different paradigms may help to understand differences in the brain dynamics of AD subjects. We hypothesized that the AD group would show lower oscillatory responses than controls after cognitive tasks, such as an oddball paradigm. Also the question of whether cholinergic applications would increase event-related delta oscillatory responses in auditory modality still needs to be addressed.

## 2. Methods

We conducted a prospective open study. Thirty-four consecutive, community-dwelling patients suffer-

Table 1
Sample characteristics

| | Controls ($n = 17$) | Alzheimer ($n = 34$) | | Group comparisons |
|---|---|---|---|---|
| | | De-novo ($n = 17$) | Medicated ($n = 17$) | $P < 0.05$ |
| Mean age (years ± SD) | 70.8 ± 5.75 | 75.1 ± 6,51 | 75.3 ± 5.68 | NS[a] |
| Gender (M/F) | 11/6 | 11/6 | 4/13 | 0.021[b] |
| Education (5–11/+11 yrs) | 1/16 | 2/15 | 2/15 | NS[b] |
| Handedness (L / R) | 10/7 | 15/2 | 13/4 | NS[b] |

[a]Kruskal-Wallis test, [b]Chi square test.

ing from dementia according to the DSM IV criteria and also with the diagnosis of probable Alzheimer's disease according to the NINCDS-ADRDA criteria [31] were included in the study. The AD group was divided into two subgroups, the medicated and the de-novo. In the medicated AD group, seventeen subjects (four males, thirteen females) were taking only cholinesterase inhibitors (ChEI) as a psychotropic agent for a duration of 3 to 6 months; and seventeen AD patients (eleven males, five females) not taking any psychotropic medication comprised the de-novo AD group. The AD groups did not differ from each other in terms of the Mini-Mental State Examination (MMSE) scores, Reisberg's Global Deterioration Scale (GDS), education, age, or handedness, as shown in Table 1. Only a gender difference was observed between the groups. The time from the onset of symptoms was between one and two years in both AD groups. The MMSE scores of all AD subjects ranged between 20 and 24, whereas those of healthy subjects were between 28 and 30 out of a possible 30 points. All of the AD subjects were on stage 4 according to the Reisberg's GDS. Nineteen healthy elderly control subjects volunteered for the study, two of whom were excluded for motor artifacts. All AD and control subjects (Table 1) underwent through a cognitive and a complete neurological, laboratory examination including blood glucose, electrolytes, liver and kidney function tests, full blood count, erythrocyte sedimentation rate, thyroid hormone, vitamin B12, HIV, VDRL. Neuroimaging (CT or MRI) were performed in all AD subjects. Presence of neuropsychiatric disease or substance abuse or serious head trauma in history were exclusionary criteria for all subjects. Healthy controls were recruited from various community sources. The study was approved by the local ethics committee. All subjects and relatives gave written informed consent.

## *2.1. Stimuli and paradigms*

The subjects' eyes were open and they were sat in a dimly-lit isolated room. Two types of stimuli were presented: simple auditory stimuli for analyzing auditory evoked oscillations and auditory oddball paradigm for analyzing auditory ERO. The auditory stimuli had a 16 ms rising time, a 50 ms falling time and a 1000 ms duration.

The auditory simple stimuli were tones of 80 dB and 1500-Hz. The inter-stimulus intervals varied randomly between 3 and 7 seconds.

A classical auditory oddball paradigm was used in the experiments. Two types of stimuli were used: task-relevant target and task-irrelevant non-target (standard). The total number of stimuli was 120 (40 target, 80 non-target). In the oddball paradigm the 80 dB, 1600-Hz tones (target) and 1500-Hz tones (non-target) were presented in a random sequence. The subjects were instructed to keep a mental count of the number of 1600-Hz tones (target). During the elicitation period of event-related oscillations, all the subjects had displayed sufficient accuracy in the mental count of the target stimuli. Patients were requested to look at the fixation point on the screen.

The maximum peak-to-peak delta responses to the target and simple auditory stimulation were analyzed and compared.

### *2.1.1. Electrophysiological recording*

EEG was recorded with 30 Ag–AgCl electrodes mounted in an elastic cap (Easy-cap) according to the international 10–20 system. Additionally, two linked earlobe electrodes (A1 + A2) served as references. The EOG from the medial upper and lateral orbital rim of the right eye was also registered. For the reference electrodes and EOG recordings, Ag-AgCl electrodes were used. All electrode impedances were less than 10 kOhm. The EEG was amplified by means of a BrainAmp 32-channel DC system machine with band limits of 0.01–250 Hz. The EEG was digitized on-line with a sampling rate of 500 Hz.

Table 2
Maximum peak-to-peak amplitudes in auditory senory oscillatory responses in Alzheimer's disease (AD) and healthy elderly controls (repeated measures of ANOVA)*

| | Controls $n = 17$ | | De-novo AD $n = 17$ | | Medicated AD $n = 17$ | |
|---|---|---|---|---|---|---|
| | mean ($\mu$V) | (SD) | mean ($\mu$V) | (SD) | mean ($\mu$V) | (SD) |
| $F_3$ | 6.73 | (2.79) | 8.64 | (0.90) | 7.01 | (1.50) |
| $F_4$ | 7.46 | (1.89) | 8.84 | (2.16) | 7.31 | (2.30) |
| $C_3$ | 7.24 | (3.39) | 7.58 | (2.63) | 6.87 | (0.54) |
| $C_4$ | 6.89 | (2.13) | 7.98 | (2.24) | 7.27 | (3.46) |
| $Tp_7$ | 4.05 | (1.63) | 3.23 | (1.97) | 3.71 | (3.63) |
| $Tp_8$ | 4.32 | (3.01) | 4.06 | (0.30) | 4.77 | (6.80) |
| $P_3$ | 4.96 | (0.21) | 5.27 | (0.41) | 6.10 | (4.05) |
| $P_4$ | 5.34 | (3.02) | 5.56 | (0.35) | 4.91 | (6.52) |

*There was no statistical significance between groups.

#### 2.1.2. Analysis

Artifacts were eliminated by manual off-line selective averaging, taking into consideration the EOG recorded from the right eye. The sweep numbers were equalized between the target, non-target and simple auditory stimulation conditions. Subject averages and grand averages were calculated for each electrode site. The data were digitally filtered in the delta (0.5–3.5 Hz) frequency range. Subsequently, we measured the maximum peak-to-peak amplitudes for each subject's averaged delta response – that is, the largest peak-to-peak value within the delta-frequency range, in terms of microvolts recorded within the time window between poststimulus 0 and 600 ms.

#### 2.1.3. Statistics

The Statistical Package for Social Studies (SPSS) program was used for statistical analysis. The differences between modalities were assessed by means of repeated measures of ANOVA. In the analysis, repeated measures of ANOVA included the between-subjects factor as group (healthy elderly controls, de-novo AD, medicated AD); and the within-subject factors as hemisphere (right, left) and location [frontal ($F_3$–$F_4$), central ($C_3$–$C_4$), temporal ($Tp_7$– $Tp_8$), parietal ($P_3$–$P_4$)]. Greenhouse–Geisser corrected p-values are reported. Post-hoc comparisons between groups were analyzed with Tukey test. The significance level was set to $p < 0.05$ for all comparisons.

## 3. Results

### 3.1. Auditory sensory evoked oscillations (EO)

No significant differences were found in delta auditory evoked oscillatory responses between groups regarding within subject factors, location or hemisphere (Table 2).

### 3.2. Auditory event-related oscillations (ERO)

The amplitudes between locations differed significantly. Repeated measures of ANOVA tests of between subjects revealed a significant difference for locations [frontal ($F_3$–$F_4$), central ($C_3$–$C_4$), temporal ($Tp_7$–$Tp_8$), parietal ($P_3$–$P_4$)] ($p < 0.0001$, df = 3.141, F = 39.742). Post-hoc paired-sample $t$ tests indicated the highest peak-to-peak amplitudes in frontal locations than all other locations ($p < 0.003$). Values at central electrode positions were also larger than those at parietal and temporal electrodes ($p < 0.002$) . Repeated measures of ANOVA tests of between subjects revealed a significant difference for groups (healthy elderly, medicated AD, de-novo AD) ($p = 0.042$, df = 2.47, F = 3.39). As shown in Table 3, the maximum amplitudes in auditory event-related delta oscillatory responses in AD and heathy elderly controls show significant differences between groups. Post-hoc Tukey analysis showed significant differences between the healthy control and medicated AD ($p = 0.049$) groups; also between healthy controls and de-novo AD ($p = 0.044$). There was no significant difference between the two AD groups. There was no significant decrease in repeated measures of ANOVA tests of between subjects for groups X locations.

We evaluated maximum peak-to-peak amplitudes within the delta frequency range, of auditory event-related oscillatory responses in AD and healthy elderly controls. Figure 1 illustrates the grand-averages of auditory event-related delta oscillatory responses to target stimuli using an oddball paradigm in three subject groups. As shown in Fig. 1 and Table 3, healthy control subjects show higher auditory ERO delta (0.5–3.5 Hz) responses to target stimuli (indicated by the blue line) than either de-novo AD ( indicated by the red line) or medicated AD ( indicated by the green line) subjects.

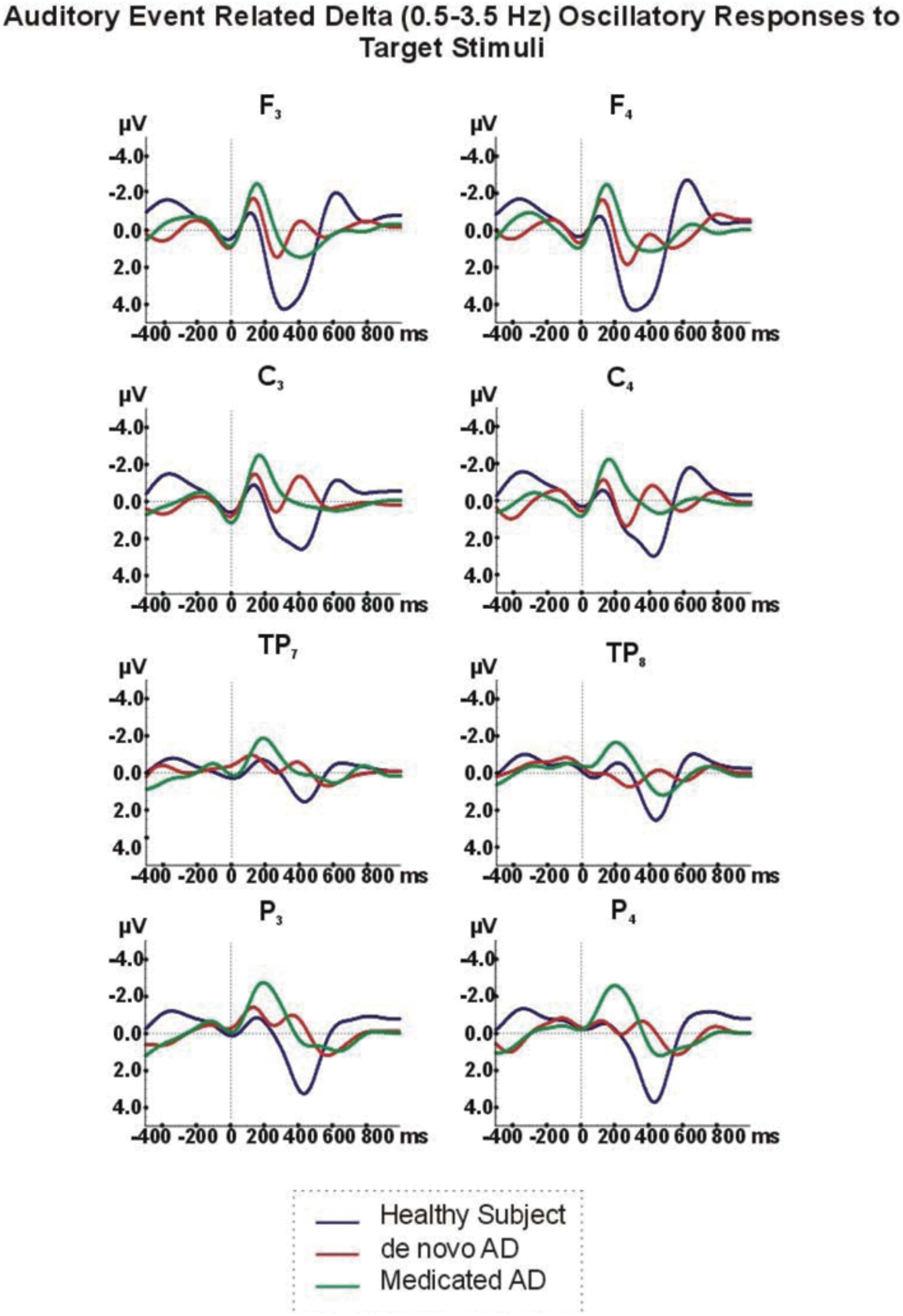

Fig. 1. Auditory event-related oscillations (ERO) delta (0.5-3.5 Hz) Responses to target stimuli in Alzheimer's disease (AD) groups and healthy elderly controls.

The fronto-central areas showed nearly 75 percent larger values than temporal regions. In frontal or central locations, delta response peak to peak amplitudes of the healthy controls may reach to 7–9 microvolts whereas they are reduced by approximately 30 percent in both groups of AD.

## 4. Discussion

Neural plasticity of brain networking provides maintenance of brain activity in healthy elderly individuals in daily life. Oscillatory brain activity as a hallmark of neuronal network function can accurately index normal

Table 3
Maximum amplitudes in auditory event-related oscillatory responses in Alzheimer's disease (AD) and healthy elderly controls (repeated measures of ANOVA)*

| Electrode | Controls $n = 17$ | | De-novo AD $n = 17$ | | Medicated AD $n = 17$ | |
|---|---|---|---|---|---|---|
| | mean ($\mu$V) | (SD) | mean ($\mu$V) | (SD) | mean ($\mu$V) | (SD) |
| $F_3$ | 9.18 | (4.77) | 6.81 | (2.53) | 6.64 | (2.26) |
| $F_4$ | 8.12 | (4.14) | 7.13 | (3.03) | 6.19 | (2.74) |
| $C_3$ | 7.86 | (3.91) | 6.11 | (2.64) | 5.55 | (2.08) |
| $C_4$ | 7.31 | (3.51) | 6.52 | (2.61) | 5.71 | (2.34) |
| $Tp_7$ | 5.26 | (2.84) | 5.04 | (3.18) | 4.68 | (2.01) |
| $Tp_8$ | 5.47 | (3.38) | 5.17 | (2.87) | 4.48 | (1.63) |
| $P_3$ | 6.94 | (3.20) | 4.84 | (1.93) | 5.53 | (2.69) |
| $P_4$ | 6.95 | (3.71) | 5.07 | (1.66) | 5.73 | (2.92) |

*Repeated measures of ANOVA test results for locations [frontal ($F_3$–$F_4$), central ($C_3$–$C_4$), temporal ($Tp_7$–$Tp_8$), parietal ($P_3$–$P_4$)] reveal $p < 0.0001$, df = 3.14, F = 39.742; and for groups (healthy elderly, medicated AD, de-novo AD), $p = 0.042$, df = 2.47, F = 3.39.

and abnormal brain functions. It provides non-invasive analysis of neuronal synchronization of firing at various frequencies [36].

Although analysis of oscillatory processes gained tremendous importance in recent years, most of work has focused on the analysis of spontaneous EEG oscillations in AD research. Summarizing the main results on spontaneous EEG addressing AD, a posterior slowing, reduced synchrony and complexity of the EEG signal can be concluded for AD patients [15]. In earlier reports, event-related potential (ERP) or P300 response was reported to be delayed in mild to moderate AD [34]. The latest reports using digital filtering of ERP, called as "event-related oscillations" may help to understand brain dynamics even further. These new neurophysiological techniques may have a potential to be a biomarker candidate, or may be used for monitoring drug effects in various clinical conditions [3,20,46]. In the present paper, we investigated auditory sensory evoked delta oscillatory responses and auditory event-related delta oscillatory responses in Alzheimer's disease. Analogously to a previous report [13], the peak-to-peak amplitudes of event-related delta oscillatory response across all groups were higher over frontal and central regions. As a group difference, we found significant changes between healthy elderly controls and AD group upon application of a cognitive task, in this case the oddball paradigm (Fig. 1 and Table 3). The de-novo and medicated AD subgroups, did not differ from each other. On the other hand, evoked delta oscillations which is elicited after a simple tone presentation without a cognitive task did not yield any group difference. This is the first study that compares the auditory sensory evoked and event-related delta oscillatory responses in subjects with AD. This comparison is important for investigating the cognitive and sensory networks in Alzheimer's disease. Also in the present study, the effects of cholinergic application were sought, but no difference between groups were observed.

Previous work by Caravaglios et al. (2008) reported a decreased event-related delta oscillatory responses upon application of oddball paradigm in AD subjects similar to ours [13]. Furthermore, their study indicated higher prestimulus single-sweep delta maximal peak-to-peak amplitudes in AD subjects. When prestimulus amplitudes diminish, a higher ratio occurs between the prestimulus and poststimulus responses, allowing more powerful cognitive responses. Therefore, the high values in prestimulus amplitudes seen in AD may correspond to less preparation for an incoming signal. This preparatory state is more likely to be correlated with frontal lobe functions [19]. Our group's earlier visual ERO studies [43,47] and movement-related cortical potential study [45] indicated poorer response over frontal or central regions in an AD group. Karrasch et al. (2006) reported significant decreases in the alpha range (7–17 Hz) at frontal, central and left temporal electrodes in their auditory ERD/ERS study [28] and Caravaglios et al. (2010) stated lack of theta enhancement during auditory oddball paradigm in AD [12].

### 4.1. *Differences between evoked and event-related delta oscillatory responses*

When the amplitudes of auditory evoked oscillatory responses are compared with those of event-related oscillatory responses, event-related oscillatory responses that is elicited after a cognitive task yields nearly six times greater amplitude of evoked oscillatory responses where only simple sensory stimulus is given [7]. In the present study, differences between groups were observed only in event-related delta oscillations, but not in evoked delta oscillations of auditory modality. The lack of group difference in EO responses may be related to the relatively smaller amplitude values elicited upon application of sensory stimuli. Another possible reason may be that the primary sensory auditory cortex is affected pathologically only in the later stages of AD, whereas heteromodal association cortices show pathological changes early in the course of disease [10]. Our subject group comprised only mild AD subjects. Sensory evoked oscillatory responses are more likely to be related to activation of primary auditory cortices, whereas event-related oscillatory responses are more likely to be related to heteromodal associative cortices. As similar to visual oscillatory responses, we propose that there exist two different auditory oscillatory networks, depending on type of stimuli (i.e. sensory or cognitive).

Our previous report on AD subjects [44] showed that visual evoked delta oscillatory responses were increased over posterior parts of hemispheres where primary and secondary visual cortices were located, whereas event-related delta oscillatory responses were diminished over fronto-central areas [43,47]. Contrary to our expectations, we did not observe the increased delta responses over temporal lobe after auditory sensory stimulation. However, Osipova et al. (2006) reported increased responses of magnetoencephalography after auditory steady state 40 Hz sensory stimulation over sensory cortex, i.e. temporal lobe, in AD [32]. As goal directed functioning requires a balance between inhibitory or excitatory inputs in the cortex, irrelevant repetitive stimulation with less cognitive load should yield lower oscillatory responses. However, decreased inhibition of cortical auditory or visual processing, possibly due to decreased prefrontal activity, may lead to increased sensory evoked cortical responses in AD. Moreover, Caravaglios et al. (2008) found decreased auditory event-related delta oscillatory responses with application of cognitive task over frontal regions in AD subjects [13]. Our previous work showing decreased visual event-related delta oscillatory responses after cognitive task and increased visual evoked delta oscillatory responses after sensory stimulation along with the work by Osipova et al. (2006) and Caravaglios et al. (2008), supports the notion of frontal lobes have inhibitory effects on sensory cortices in Alzheimer's disease [11,18,32].

### 4.2. *Delta frequency range of ERO in AD*

Auditory evoked delta oscillatory responses in AD subjects is similar to those of healthy subjects, however auditory event-related delta oscillatory responses of same modality in AD indicate significant decrease in comparison to healthy subjects. Although, the medicated AD group had a higher ratio of females to males, we believe that lower delta ERO responses cannot be explained by differing gender balance between groups in the present study, as Güntekin and Başar (2007) showed that female subjects displayed higher values of delta ERO responses [21].

Delta response decrease is a common feature of visual and auditory ERO in AD. Also, cholinergic medication effects cannot be readily observed in delta ERO in both modalities. Our results favor the hypothesis that neural delta networks are activated during cognitive tasks and the reduced delta response is a general phenomenon in patients with AD, due to cognitive impairment. Delta ERO responses seem to be modality-independent and resistant to cholinergic treatment.

### 4.3. *Delta oscillatory responses have a functional role in brain dynamics*

We used the oddball paradigm with event-related stimulation, since it is known that AD subjects are cognitively impaired. Demiralp et al. (2001) reported that, after application of stimulus with decreasing intensities, the oscillatory responses occur nearly in pure delta range when stimuli get closer to subjective threshold [16]. At the threshold intensity, stimulation focuses the attention of the subject. In this context, the lower delta oscillatory responses in AD subjects upon either visual or auditory oddball paradigm are quite understandable, as individuals with AD tend to show decreased attentional or decision-making focus. We believe event-related delta oscillatory responses are very important parameters for differentiating AD from healthy controls, regardless of their taking cholinergic medication. Also further investigations using phase-locking or prestimulus/poststimulus response analyses can provide more information regarding changes in brain dynamics in AD.

### 4.4. *Comparison of visual and auditory oscillatory responses*

Our earlier findings on visual oscillatory dynamics in AD subjects can be summarized as follows: 1) visual evoked delta oscillations in AD subjects show increased maximum amplitudes ranges over posterior hemispheres where visual sensory cortices are located (15) [44]; 2) visual event-related delta oscillatory responses in AD subjects show significant decreased maximum amplitudes over central areas [43]; and 3) visual coherence elicited after sensory stimulus indicates reduced functional connectivity between a fronto-occipital electrode pair, whereas coherences after cognitive task (oddball paradigm) show decreased values between frontal and all other cortical areas [23]. This finding once more implies that there exist at least two different networks (i.e. sensory and cognitive), and that application of a cognitive task causes a more widespread activation of neural networks [46]. The coherence values indicate neuronal synchronization and connectivity, and they almost double after cognitive task in relation to those elicited after simple sensory stimulus [4].

As a conclusion, auditory event-related delta oscillatory responses yield lower maximum peak-to-peak amplitudes in AD subjects, as seen similar in visual event-related delta oscillatory responses. Event-related delta oscillatory responses in either auditory or visual modalities appear to be resistant to cholinergic medication. Cognitive tasks are more useful than simple sensory stimulation in highlighting the difference between groups, as the maximum amplitudes of event-related delta oscillatory responses may be double the level of those elicited after sensory stimulation.

The comparison, as discussed in the previous section, shows that there are several parameters manifesting electrophysiological differences between AD and healthy subjects. The relatively small sample size of the present study does not yet permit a definitive conclusion; however, it can be assumed that with larger studies, there is a potential to use the introduced ensemble of parameters as biomarkers. This electrophysiological ensemble of parameters allows the analysis of brain dynamics within the time window of 600 ms poststimulus, thus serving as a dynamic indicator that is impossible to reach by any other functional neuroimaging methods with a lower time resolution, such as fMRI or PET. Further studies should aim not only to separate the physiological and pathological states of the brain at the group level, but should also consider analysis at the individual level. In order to accomplish this task, there is a great need for the use of biological markers, together with neurophysiological techniques that can provide inexpensive and non-invasive methods of screening for brain pathological conditions.
